# Supplementary material for: Cardiologist-Performed Baseline Evaluation with an Assessment of Coronary Status for Prostate Cancer Patients Undergoing Androgen Deprivation Therapy: Impact on Newly Diagnosed Coronary Artery Disease
Source: Cancers (Basel). 2023 Aug 17;15(16):4157. doi: 10.3390/cancers15164157 (PMC10452606; doi:10.3390/cancers15164157)
Supplement: Supplementary file 1 [file cancers-15-04157-s001.zip › cancers-2467346-supplementary.pdf]

Supplementary Materials

Figure S1: Flowchart

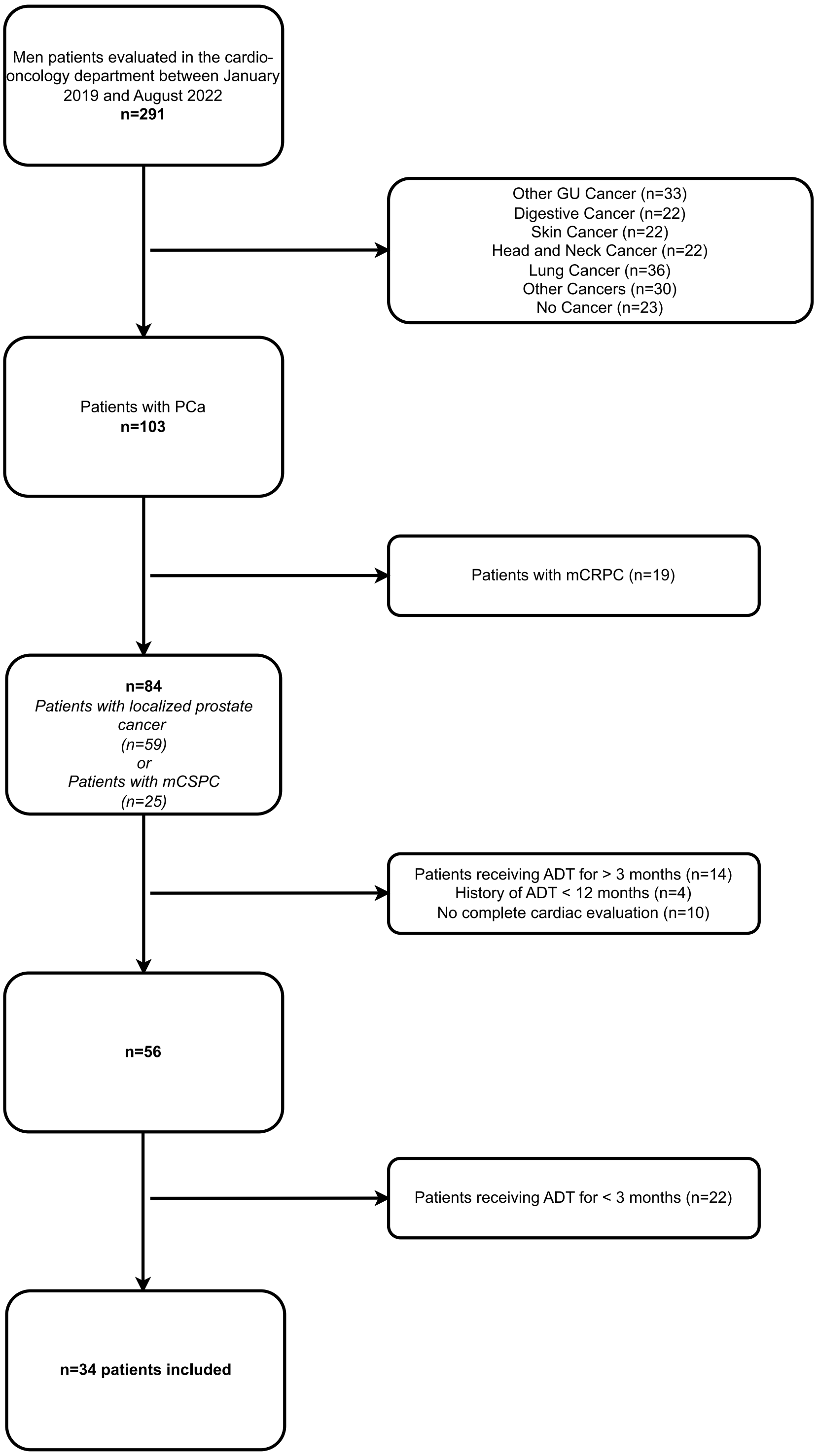

GU:Genito-urinary; PCa: Prostate Cancer; mCRPC: metastatic Castration-Resistant Prostate Cancer; mCSPC: metastatic Castration-Sensitive Prostate Cancer; ADT: Androgen Deprivation Therapy

Table S1: Results of cardiac stress tests

	Total (N=27)
**Type Cardiac Stress Test**	
Cardiac Stress Test (TTE)	25 (92.6%)
Cardiac Scintigraphy	2 (7.4%)
**Watts Theorical Target (%)**	
Mean (SD)	101 (17.0)
Median [Min, Max]	101 [58.0, 135]
Missing	3 (11.1%)
**Cardiac Frequency Theorical Target (%)**	
Mean (SD)	84.1 (20.1)
Median [Min, Max]	87.0 [0, 108]
Missing	2 (7.4%)
**Clinical Symptoms**	
No	27 (100%)
Yes	0 (0%)
**ECG modifications**	
No modifications	22 (81.5%)
Ventricular excitability	2 (7.4%)
Ischemia	2 (7.4%)
Ventricular and supraventricular excitability	0 (0%)
Missing	1 (3.7%)
**Number of dyskinetic segment**	
0	18 (66.7%)
1	3 (11.1%)
2	4 (14.8%)
3	2 (7.4%)
